# Successful balloon fixation technique to prevent dislocation of a fully covered self-expanding metal stent for biliary stricture

**DOI:** 10.1055/a-2515-3156

**Published:** 2025-01-31

**Authors:** Kosuke Takahashi, Eisuke Ozawa, Yasuhiko Nakao, Masanori Fukushima, Hisamitsu Miyaaki, Kazuhiko Nakao

**Affiliations:** 1 Department of Gastroenterology and Hepatology, Graduate School of Biomedical Sciences, Nagasaki University, Nagasaki, Japan; 2 Department of Gastroenterology and Hepatology, Sasebo City General Hospital, Sasebo, Japan

Fully covered self-expanding metal stents (FCSEMSs) are commonly used to manage benign biliary strictures 1 . However, stent migration and dislocation during and after endoscopic retrograde cholangiopancreatography (ERCP) remain a challenge 2 3 4 5 . Stent dislocation rarely occurs when the delivery system catches on the stent during removal. This case report demonstrates the successful prevention of stent dislocation using a balloon catheter inserted through the percutaneous transhepatic biliary drainage (PTBD) route.

An 83-year-old man who had undergone living-donor liver transplantation was referred to our hospital to address an anastomotic biliary stricture (ABS) and to extract intrahepatic stones. Computed tomography revealed intrahepatic bile duct dilatation secondary to hepatolithiasis ( Fig. 1 **a** ). Although endoscopic retrograde cholangiography confirmed the presence of intrahepatic stones, stone removal was not possible because of severe ABS ( Fig. 1 **b** ). The patient subsequently underwent PTBD, and the stones were successfully removed from the common bile duct using electrohydraulic lithotripsy via the PTBD route ( Fig. 1 **c** ).

**Fig. 1:**
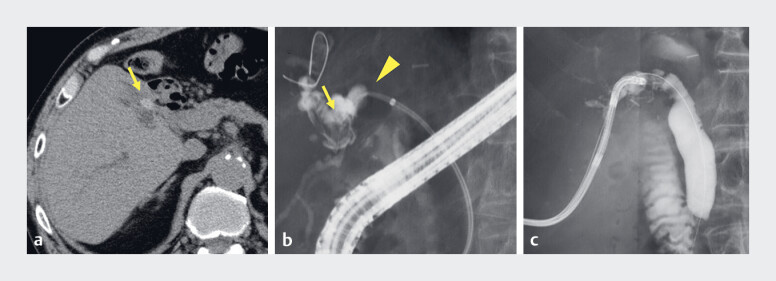
Computed tomography and cholangiography images. **a** Computed tomography showed intrahepatic bile duct dilatation due to hepatolithiasis (arrow). **b** Cholangiography showed severe anastomotic biliary stricture (arrowhead) and intrahepatic stones (arrow). **c** Electrohydraulic lithotripsy was performed through a percutaneous transhepatic biliary drainage route.

Subsequently, insertion of an FCSEMS (BonaStent M-intraductal; Standard Sci Tech, Seoul, South Korea) was attempted to improve the ABS. During removal of the delivery system, the stent dislodged because the catheter tip became caught on the stent ( Fig. 2 **a, b** ). To prevent stent dislocation, a balloon catheter was inserted through the PTBD route, positioned at the proximal end of the metal stent, and inflated. This technique successfully stabilized the stent, allowing safe removal of the delivery system without further stent displacement ( Fig. 2 **c, d** , Video 1 ). No adverse events, including stent dislocation, were observed after the procedure ( Fig. 2 **e** ).

**Fig. 2:**
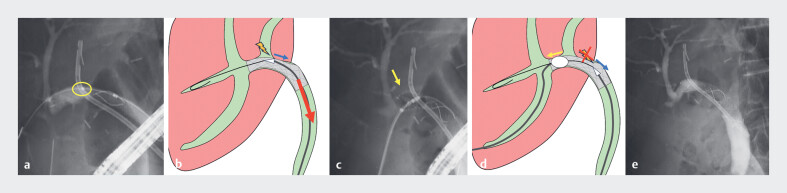
Cholangiography and schematic diagrams. **a, b** The delivery system could not be removed after metal stent deployment (circle). **c, d** A balloon catheter was used to stabilize the stent and prevent dislocation during removal of the delivery system (arrow). **e** Cholangiography showed successful metal stent placement.

Balloon fixation technique to prevent dislocation of the metal stent.Video 1

This case demonstrates that using a balloon catheter via the percutaneous route can effectively prevent stent dislocation during ERCP when the delivery system is caught on the stent. This straightforward yet effective technique may be useful in preventing stent dislodgment during challenging biliary interventions.

Endoscopy_UCTN_Code_CPL_1AK_2AZ

## Notes

Endoscopy E-Videos https://eref.thieme.de/e-videos
*E-Videos* is an open access online section of the journal *Endoscopy* , reporting on interesting cases and new techniques in gastroenterological endoscopy. All papers include a high-quality video and are published with a Creative Commons CC-BY license. Endoscopy E-Videos qualify for HINARI discounts and waivers and eligibility is automatically checked during the submission process. We grant 100% waivers to articles whose corresponding authors are based in Group A countries and 50% waivers to those who are based in Group B countries as classified by Research4Life (see: https://www.research4life.org/access/eligibility/ ). This section has its own submission website at https://mc.manuscriptcentral.com/e-videos .
